# Electroosmotic Flow-Based Nanoinjection Technique Using a Nanopipette for Green Microalgae

**DOI:** 10.1007/s10126-025-10487-0

**Published:** 2025-07-01

**Authors:** Tsuyoshi Tanaka, Kaoruko Akasaka, Rein Yasui, Naoto Shinohara, Tomoko Yoshino, Daisuke Nojima, Makoto Mochizuki, Takatoshi Ohata, Fumitaka Kamachi, Tsuneji Sawai

**Affiliations:** 1 Division of Biotechnology and Life Science, Institute of Engineering, Tokyo University of Agriculture and Technology, 2-24-16 Naka-Cho, Koganei, Tokyo 184-8588 Japan; 2 Yokogawa Innovation Switzerland GmbH, Hegenheimermattweg 167A, 4123 Allschwil, Switzerland; 3 Yokogawa Electric Corporation, 2-9-32 Naka-Cho, Musashino-Shi, Tokyo, 180-8750 Japan

**Keywords:** Nanoinjection, Microalgae, Electroosmotic flow, Nanopipette

## Abstract

Microalgae are gaining attention as promising candidates for CO₂ fixation and biomaterial production due to their non-competition with food and feed resources, as well as their high growth rates and photosynthetic efficiency. To expand their application as hosts for biomaterial production, it is essential to develop efficient and versatile gene modification technologies for microalgal metabolic engineering. Achieving this requires the establishment of an effective and broadly applicable material delivery method across diverse microalgal species. In this study, we developed a novel nanoinjection technique for single microalgal cells, utilizing electroosmotic flow through a nano-sized pipette (nanopipette). This nanopipette enables precise, automated delivery of solutions into cells at the femtoliter scale. Optimum injection conditions, including cell morphology, injection voltage, and injection time, were identified using fluorescein isothiocyanate (FITC)-labeled dextran in two green microalgae species, *Haematococcus* sp. and *Tetraselmis* sp. The method achieved injection efficiencies of 44% for *Haematococcus* sp. and 45% for *Tetraselmis* sp. This technique demonstrates substantial potential for efficient genome editing and subsequent metabolic engineering in a wide range of microalgae species.

**Supplementary Information:**

The online version contains supplementary material available at 10.1007/s10126-025-10487-0.

## Introduction

Microalgae have emerged as promising hosts for the production of fine chemicals, including polyunsaturated fatty acids (PUFAs), terpenoids, and carotenoids (Chen et al. 2023; Huang et al. 2021). A key advantage of using microalgae for fine chemical production lies in their CO₂ fixation ability through photosynthesis. Advances in genetic engineering have not only enhanced the productivity of existing fine chemicals (Sreenikethanam et al. 2022) but have also enabled the biosynthesis of novel compounds through metabolic engineering (Wichmann et al. 2018; Maeda et al. 2021; Tanaka et al. 2021). Consequently, numerous genetic modification techniques have been developed for microalgae.

Despite these advancements, the efficient genetic transformation of microalgae remains a major challenge. Current transformation techniques, such as microparticle bombardment or electroporation, have been used for various eukaryotic microalgae (Wang et al. 2024). However, these methodologies generally suffer from low transformation efficiencies (Wang et al. 2019; Run et al. 2016) compared to other hosts, such as bacteria (e.g., *Escherichia coli*) and yeasts (e.g., *Saccharomyces cerevisiae*). In green microalgae, the transformation efficiencies ranged from 7.6 × 10 transformants per µg-DNA to 1.8 × 10^4^ transformants per µg-DNA (Kumar et al. 2018). In contrast, the transformation efficiency in *E. coli* was 8.7 × 10⁸ transformants per µg-DNA (Heiat et al. 2012). Furthermore, the range of genetically modifiable microalgae is limited to specific genera and species.

The primary barrier to efficient genetic transformation in microalgae is the presence of the cell wall, which significantly hinders the intracellular delivery of nucleic acids and proteins. For example, previous studies using a cell wall-deficient strain of *Chlamydomonas reinhardtii* have demonstrated that large molecules are less likely to cross the cell wall by electroporation (Jeon et al. 2013; Muñoz et al. 2018). To overcome these problems, microinjection is a promising approach for intracellular delivery, enabling the direct insertion of nucleic acids, proteins, or chemicals into cells using a glass capillary. This technique involves the physical penetration of the cell membrane or cell wall, allowing the precise delivery of materials. Chen et al. (2022) introduced CRISPR-Cas9 ribonucleoprotein (RNP) complexes into *Euglena gracilis* through microinjection, achieving gene knockout with a 15-fold higher efficiency than that achieved using electroporation. However, this achievement can be attributed to the use of a cell wall-less mutant. These results suggest that microinjection into cell wall-bearing microalgae remains a considerable challenge. Thus far, there have been no reports of the successful microinjection or nanoinjection of target molecules into microalgae with cell walls.

In this study, we developed a novel nanoinjection technique for single-microalgal cells using a nano-sized capillary, referred to as a nanopipette. Previously, nanopipettes have been utilized for the collection of mRNAs from single cells via electrochemical flow-based aspiration (Stanley et al. 2023). For our study, nanopipettes with a diameter of approximately 30 nm were employed to enable precise and automated solution delivery into cells at the femtoliter scale, based on electroosmotic flow. We evaluated the performance of this nanoinjection system using fluorescein isothiocyanate (FITC)-labeled dextran in two green microalgae species: *Haematococcus* sp., a well-known producer of astaxanthin (Mularczyk et al. 2020), and *Tetraselmis* sp. identified as a glycosyl ceramide producer (Arakaki et al. 2013). Our proposed method demonstrates significant potential for efficient genetic transformation, including genome editing and subsequent metabolic engineering, across a diverse range of microalgae species.

## Materials and Methods

### Strains and Culture Conditions

Green microalgae strains from the marine microalgal culture collection of our university were used in this study. *Haematococcus* sp. strain NKG7001 (DDBJ/EMBL/GenBank databases accession no. LC856495) and *Tetraselmis* sp. strain NKG400013 (Arakaki et al. 2013) were cultured in BG-11 medium and IMK medium (Nihon Pharmaceutical Co., Tokyo, Japan), respectively, at 25 °C under continuous illumination (photon flux density: 15 µmol/m^2^/s). Both media were supplemented with ampicillin (50 µg/ml) to prevent bacterial contamination. For control experiments, human embryonic kidney (HEK293T) cell lines (ATCC® CRL 3216™) were utilized. These cells were cultured in DMEM medium (Thermo Fisher Scientific, Tokyo, Japan) supplemented with 10% fetal bovine serum (FBS; Invitrogen, Carlsbad, CA) and 1% penicillin–streptomycin (Invitrogen). HEK293T cells were maintained at 37 °C in a 5% CO₂ atmosphere and subcultured every 3–4 days to ensure optimal growth conditions.

### Preparation of Cell Samples for Nanoinjection

Microalgal cells were prepared on agarose gel pads for microscopic observation and nanoinjection. Low melting point agarose (1.5–2.0% w/v) dissolved in growth medium containing ampicillin (50 μg/ml) and kanamycin (50 μg/ml) was used as the substrate for cell preparation. For *Haematococcus* sp., a 10–15 μl suspension of cells was seeded onto a 3.5 cm Petri dish and air-dried to allow water evaporation. For *Tetraselmis* sp., a 10 μl cell suspension was pipetted onto an 18 mm square glass coverslip. Subsequently, 200 μl of agarose solution was layered on top and allowed to solidify at room temperature. The solidified gel pad was carefully peeled off the coverslip for further processing. For HEK293T cells, a suspension was seeded onto a 3.5 cm dish and incubated for 24 h to allow cell adhesion. The attached cells were then used directly for nanoinjection.

#### Nanoinjection System

**Fig. 1:**
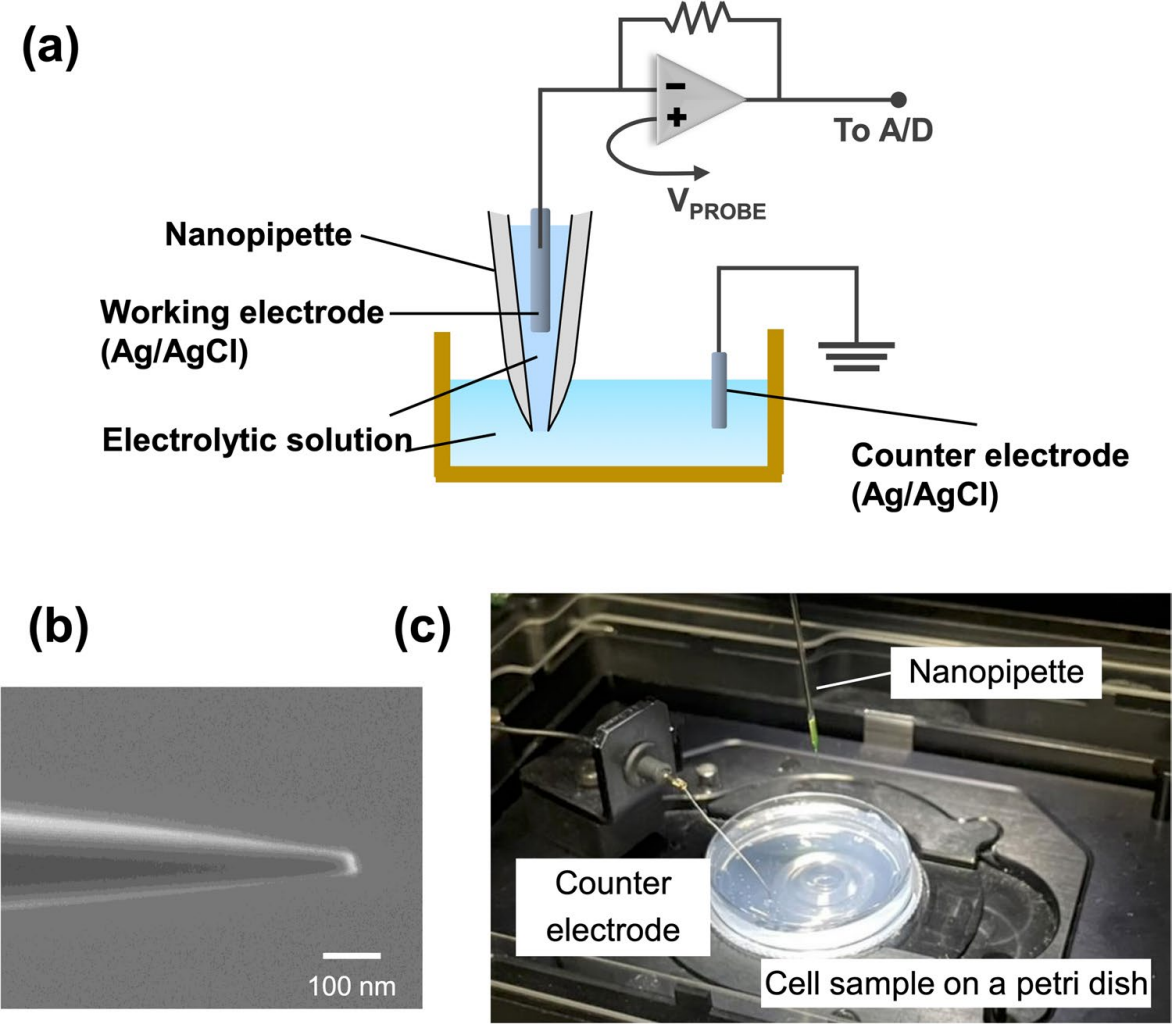
(**a**) Overview of the nanoinjection system (Single Cellome™ Unit SU10). (**b**) Scanning electron microscopy (SEM) image of the nanopipette tip. Scale bar = 100 nm. (**c**) Experimental setup for the nanoinjection process

The Single Cellome™ Unit SU10 (Yokogawa Electric Corporation, Tokyo, Japan), integrated with an inverted microscope (IX83, Olympus, Tokyo, Japan), was employed for nanoinjection (Fig. 1a). Nanopipettes (SU10ACC-NP02, Yokogawa Electric Corporation) were constructed from quartz capillaries with tip diameters in the nanometer range. The nanopipette was filled with phosphate-buffered saline (PBS; 1 mM KH₂PO₄, 155 mM NaCl, 3 mM Na₂HPO₄·7H₂O, pH 7.4; Thermo Fisher Scientific, Tokyo, Japan) (Stanley et al. 2023) (Fig. 1b). A 4 μl solution of the injection reagent was loaded into the nanopipette, and centrifuged at 2,000 × *g* for 1 min to ensure the reagent filled the pipette tip. An Ag/AgCl electrode served as the working electrode, while a second Ag/AgCl wire functioned as the counter electrode (Fig. 1c). The SU10 controller automated the nanopipette’s movement for targeting and penetrating individual cells. For ionic current measurement, the Ag/AgCl electrodes were positioned within the nanopipette and the sample solution to monitor real-time current changes during operation.

### Nanoinjection of Fluorescent Dye into Microalgae Cells

**Fig. 2:**
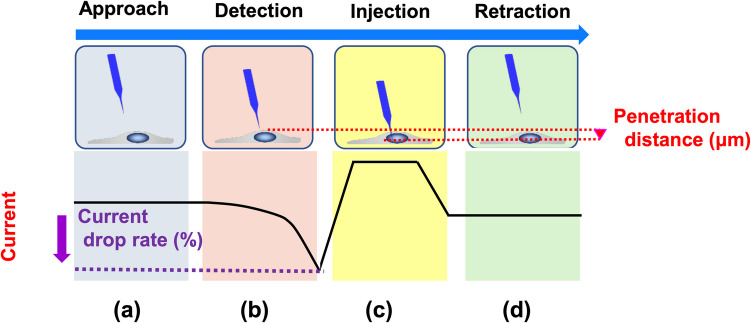
Changes in current observed during each operational stage of the nanopipette

FITC-labeled dextran (10 mg/ml, MW: 70,000; Merck, Darmstadt, Germany) dissolved in PBS was used as the injection reagent. Microalgal cells immobilized on agarose gel pads were immersed in 3 ml PBS within a Petri dish. The nanopipette was first manually positioned approximately 50 μm above the target cell under microscopic observation, based on the focal position of the cell surface. Automated injection was subsequently performed to deliver the fluorescent dye into the cell. Voltage was applied to the electrodes to monitor ionic currents during the procedure. When the nanopipette reached the cell surface (Fig. 2a), a decrease in ionic current occurs due to ion migration hindrance from cell surface, indicating the proximity of the cell surface (Fig. 2b). Surface detection was defined as a current signal reduction between 7 and 20% based on pre-experiments. After detecting the surface, the nanopipette was moved down such that it penetrates the cell to a predetermined depth (≤ 10 µm for *Haematococcus* sp. and ≤ 8 µm for *Tetraselmis* sp.). Fluorescent dye was injected via electroosmotic flow generated by applying voltage (Fig. 2c). Each nanopipette was continuously used for the injection of approximately 50 single cells, which were referred to as trial cells. Successful dye delivery was confirmed using fluorescence microscopy, and the injection rate was calculated as the ratio of successfully injected cells to the total number of trial cells. After the injection, the nanopipette was retracted from the cell (Fig. 2d). The injected cells in the Petri dish were washed with culture medium and cultured at 25 °C in fresh medium for two weeks. The cell division was confirmed by microscopy after a 4-day cultivation. The same injection protocol was applied for HEK293T cells, using the following parameters: an applied voltage of 5 V; a penetration depth of 2 μm; and an injection duration of 0.5 s. Surface detection in HEK293T cells was defined as a 23% reduction in the current signal.

## Results

### Monitoring Ionic Current During Nanoinjection into Microalgae

**Fig. 3:**
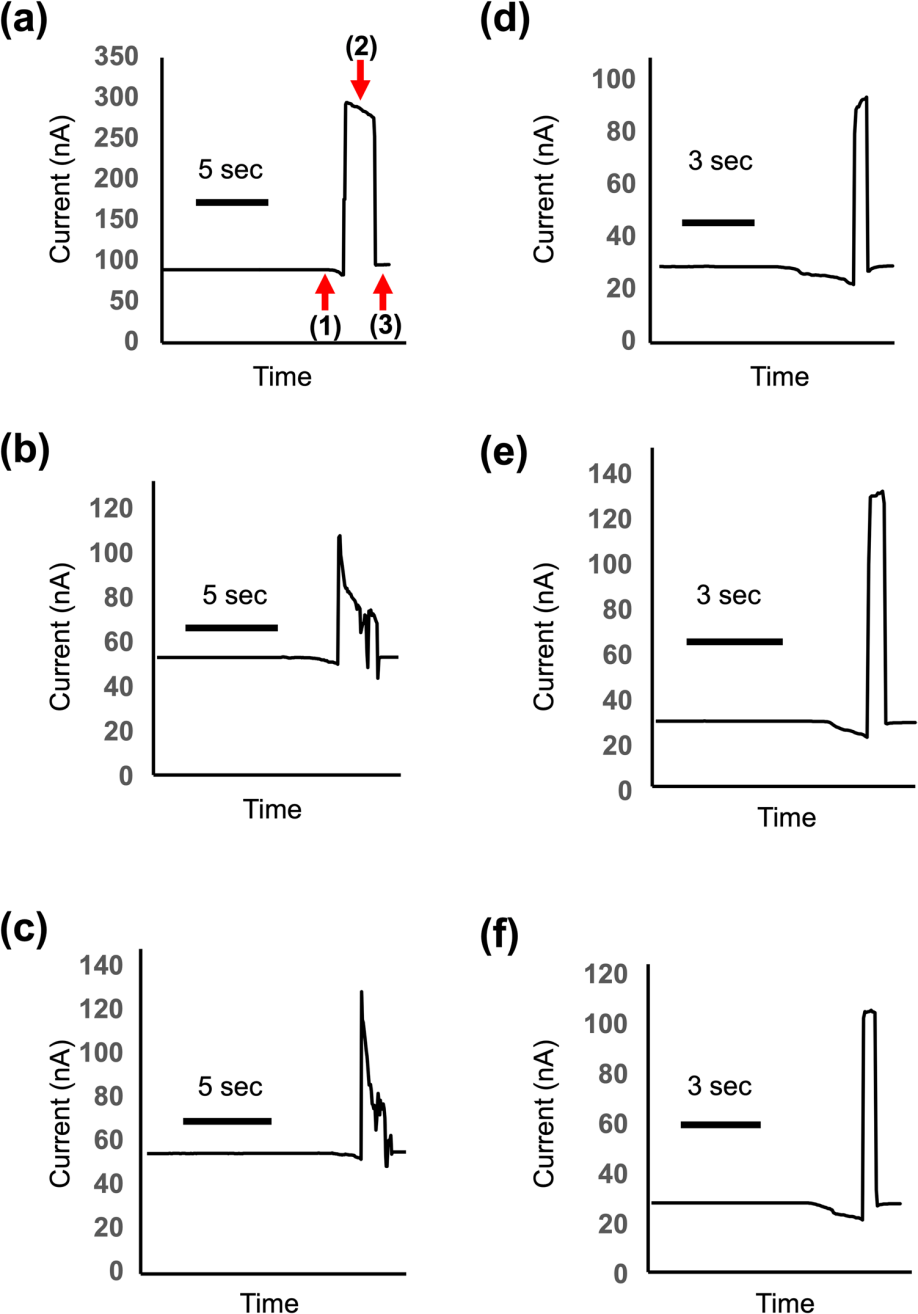
Current changes recorded during the nanoinjection of FITC-dextran into (**a**–**c**) green microalga *Haematococcus* sp. and (**d**–**f**) HEK293T cells

Figure 3a illustrates a typical ionic current change observed during nanoinjection into the green microalga *Haematococcus* sp. A distinct current drop, marked by arrow (1) in Fig. 3a, was detected, indicating the nanopipette’s contact with the cell surface. Subsequently, the nanopipette was automatically advanced to penetrate the cell surface, and a preset voltage was applied to facilitate the introduction of FITC-dextran. This step resulted in a current peak corresponding to the applied potential (Fig. 3a; arrow (2)). Upon completion of the FITC-dextran injection, the nanopipette was retracted, and the current value returned to its original state (Fig. 3a; arrow (3)). However, ionic current patterns during nanoinjection into microalgae displayed high variability (Fig. 3a-c) compared to the more consistent patterns observed in mammalian cells (Fig. 3d-f). Despite this variability, via fluorescence microscopy, we confirmed the successful nanoinjection of the fluorescent dye in all experiments presented in Fig. 3. The observed variability is likely attributable to the presence of cell walls in microalgae, which may interfere with ion current measurements.

**Fig. 4:**
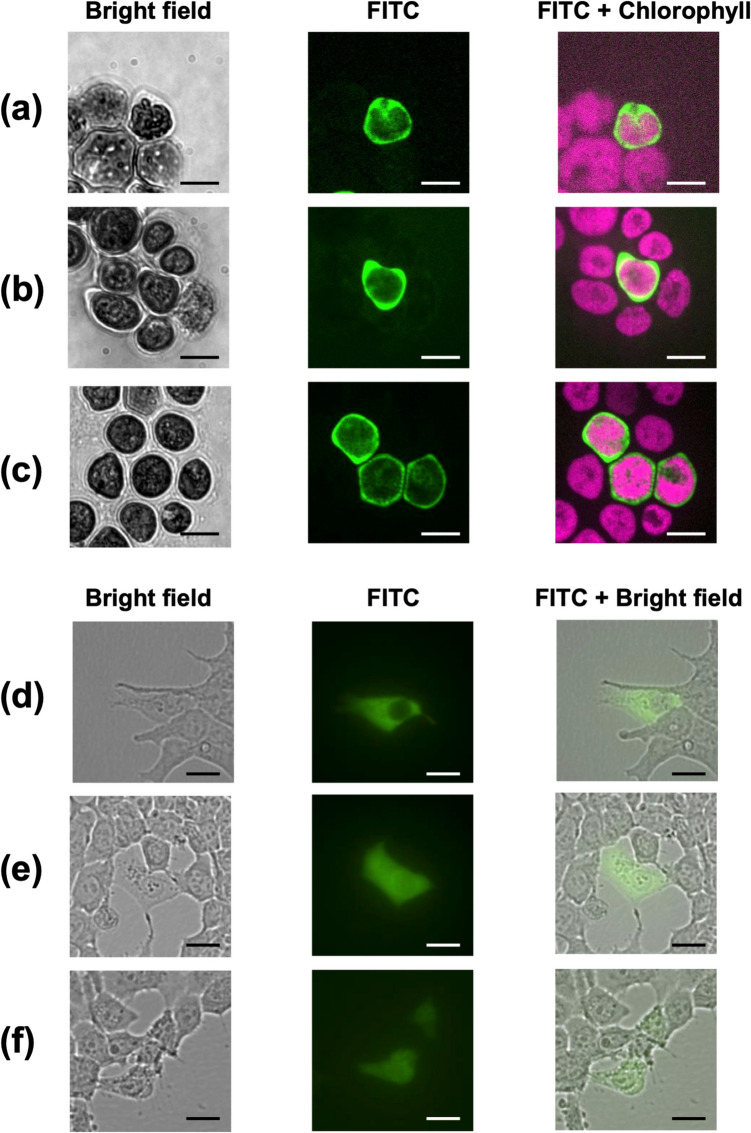
Microscopic images following the injection of FITC-dextran: (**a**–**c**) Microalgal cells (*Haematococcus* sp.) and (**d**–**f**) mammalian cells (HEK293T). Scale bars = 20 μm. Green: FITC, Magenta: Chlorophyll autofluorescence

Figure 4 presents the fluorescence microscopy images of microalgal cells after the injection of FITC-dextran. Notably, the localization of FITC-dextran differed between algal and mammalian cells. In mammalian cells, fluorescence was evenly distributed throughout the cytoplasm (Fig. 4d-f), whereas in microalgae, FITC-dextran localization was predominantly confined to regions surrounding chloroplasts (Fig. 4a-c). This restricted distribution in microalgae can be attributed to their limited cytoplasmic space, as chloroplasts occupy the majority of the cell volume. Furthermore, the efficiency of FITC-dextran delivery into microalgae was inconsistent, contrasting with the high success rate observed in mammalian cells (25 injected cells/27 trial cells; 92.6%). These differences suggest that the unique surface properties of microalgal cells, particularly the presence of cell walls, significantly influence the nanoinjection process. To better understand these effects, subsequent experiments were conducted to evaluate the impact of the microalgal growth stage on injection efficiency.

### Injection Rates in *Haematococcus* sp. with Different Cell Morphologies

**Fig. 5:**
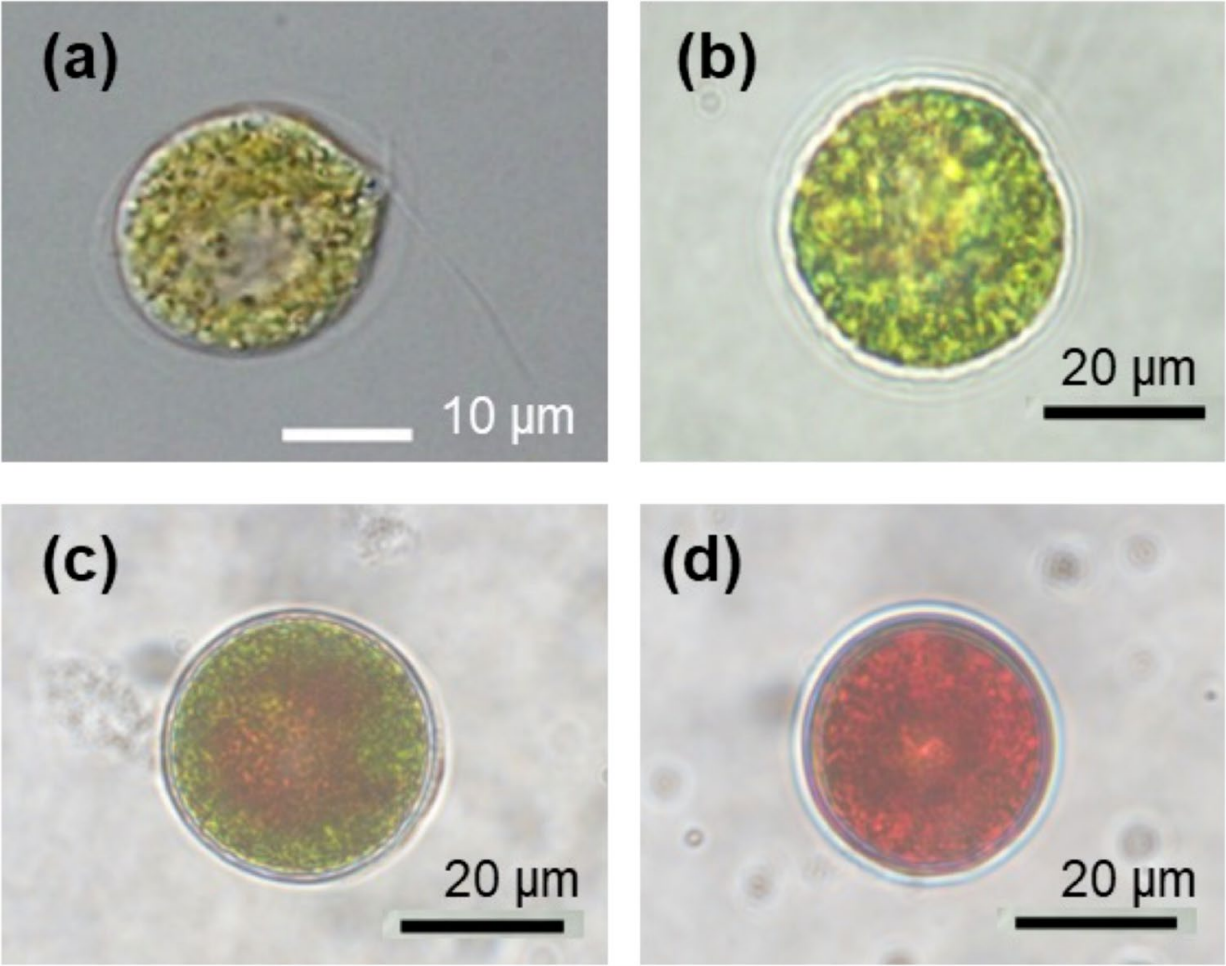
Phase-contrast microscopic images of *Haematococcus* sp. strain NKG7001 at different cell stages: (**a**) Flagellated cell, (**b**) palmelloid, (**c**) intermediate cell, and (**d**) cyst

The green microalga *Haematococcus* sp. undergoes significant morphological transitions throughout its life cycle, progressing through four distinct stages: flagellated cells, palmelloids, intermediate cells, and cysts (Fig. 5) (Shah et al. 2016). As the cells advance toward the cyst stage, they begin to accumulate astaxanthin, resulting in a red coloration. Concurrently, their cell wall structure undergoes notable changes, becoming progressively thicker and more rigid. These structural changes, particularly cell wall thickening, were anticipated to influence the efficiency of nanoinjection. To assess the impact of these morphological variations, the injection rates of FITC-dextran were evaluated across the different cell types (Table 1). Successful injections were achieved in flagellated cells and palmelloids, with injection rates of 21% for flagellated cells and 6% for palmelloids. In contrast, no successful injections were observed in intermediate cells or cysts, likely due to the increased thickness and rigidity of their cell walls during these later developmental stages. As a result, flagellated cells and palmelloids of *Haematococcus* sp. were selected for subsequent experiments due to their higher injection efficiency.

**Table 1:** Injection rates of the nanoinjection system in *Haematococcus* sp. with different cell morphologies

Cell morphology	Flagellated cells	Palmelloids	Intermediate cells	Cysts
Injection rate (%)	21	6	0	0
Injected cells/Trail cells	7/33	1/17	0/20	0/30

### Optimum Conditions for Nanoinjection into Green Microalgae

To optimize nanoinjection into *Haematococcus* sp., the applied voltage and injection duration were systematically evaluated (Table 2(a-b)). The highest injection efficiency, 44%, was achieved at an applied voltage of 6 V with an injection time of 1 s. Lower injection efficiencies at reduced voltages (2 V and 4 V) were likely due to insufficient electroosmotic flow, which hindered adequate delivery of FITC-dextran into the cells. Furthermore, successful nanoinjection of FITC-dextran was confirmed at injection durations of 0.1 s or longer (Table 2(b)).

**Table 2:** Optimization of (a) Applied voltage and (b) Injection time for nanoinjection into *Haematococcus* sp.

(a)					
Applied voltage (V)	2	4	6	8	10
Injection rate (%)	20	13	44	17	3
Injected cells/Trial cells	4/20	4/30	37/85	4/23	1/40
(b)					
Injection time (s)	2.00	1.00	0.50	0.10	0.05
Injection rate (%)	33	44	30	16	0
Injected cells/Trial cells	13/40	37/85	18/60	9/55	0/33

A similar optimization process was conducted for *Tetraselmis* sp. cells. As with *Haematococcus* sp., nanoinjection was successfully achieved under all tested conditions when the injection time was fixed at 1 s (Table 3(a)). The optimal conditions for *Tetraselmis* sp. were identified as an applied voltage of 5 V with a 1-s injection time, yielding an injection efficiency of 45%. Fluorescence signals were detected in *Tetraselmis* sp. cells (Fig. S1) only when the injection time was 0.1 s or longer (Table 3(b)). To evaluate the impact of nanoinjection on cell viability, the division of injected cells was observed under a microscope. Among 30 injected cells, 17 cells (57%) showed successful division, indicating that the nanopipette-based nanoinjection causes minimum cellular damage.

**Table 3:** Injection rates of the nanoinjection system at different injection conditions for *Tetraselmis* sp.

(a)					
Applied voltage (V)	2	4	5	6	
Injection rate (%)	3	28	45	28	
Injected cells/Trial cells	1/40	11/40	18/40	11/40	
(b)					
Injection time (s)	2.00	1.00	0.50	0.10	0.05
Injection rate (%)	33	44	30	16	0
Injected cells/Trial cells	13/40	37/85	18/60	9/55	0/33

## Discussion

A novel nanoinjection technique for microalgal cells was developed using a nanopipette that leverages electroosmotic flow. In this study, two green microalgae species, *Haematococcus* sp. and *Tetraselmis* sp., were used to evaluate the method. The optimized injection efficiencies of FITC-labeled dextran (70 kDa) were nearly identical: 44% for *Haematococcus* sp. (Table 2) and 45% for *Tetraselmis* sp. (Table 3). These results suggest that the nanoinjection technique may achieve similar efficiencies in other green algae species. These values could be substantially higher than those of conventional injection methods. In the injection of bovine serum albumin (66.5 kDa) by electroporation, the percentages of injected cells were 1.3% for *Chlorella vulgaris*, 2.7% for *Acutodesmus obliquus*, and 1.7% for *Neochloris oleoabundans* (Muñoz et al. 2018). As noted in the Introduction, the presence of a rigid cell wall is recognized as a major barrier to efficient genetic transformation in microalgae. The relatively high delivery efficiencies achieved in the present study suggest that nanoinjection may serve as a promising tool to address the above issue and facilitate more effective transformation techniques in microalgae.

A key advantage of the nanoinjection technique is its precise control over injection volume, which can be finely tuned by adjusting the injection voltage and time. When FITC-dextran was introduced into mammalian cells, fluorescence intensity increased proportionally with injection times (Fig. S2), demonstrating that nanoinjection enables quantitative delivery. Precise control is particularly beneficial for applications such as plasmid or Cas9 protein introduction. The injection process proceeded automatically at approximately one cell per minute with each nanopipette capable of performing up to 50 consecutive injections. Further improvements in throughput may be achieved by incorporating automated cell recognition and positioning systems, potentially enabling large-scale applications without compromising precision.

Traditionally, DNA transfer into microalgal cells has been achieved using microparticle bombardment, which forces DNA into cells by coating it onto gold or tungsten microparticles. While effective across a broad range of microalgae, this method often results in physical damage to cells, leading to low transformation efficiencies. Additionally, transferred DNA is frequently fragmented and integrated at random genomic sites. Consequently, there has been a growing demand for alternative, less invasive methods. Recently, bacterial conjugation has emerged as a promising approach for genetic transformation in microalgae (Karas et al. 2015; Garza et al. 2023). This method enables minimally invasive DNA introduction, with plasmid DNA being transferred to and maintained in microalgal cells without fragmentation (Kassaw et al. 2022; Garza et al. 2023). Bacterial conjugation relies on a conjugative pilus—a protein assembly approximately 8 nm in diameter—that acts as a conduit for transferring DNA between cells (Frost et al. 1994). The nanoinjection technique developed in this study shares similarities with bacterial conjugation in that both provide minimally invasive DNA transfer, suggesting that nanoinjection could offer an alternative to current transformation strategies.

In the nanoinjection system, ionic currents during the injection remain almost constant (Fig. 2c), as observed in mammalian cells (Fig. 3a, d-f). However, in the case of microalgae, injection peaks were often seen, and then, signal fluctuations were detected (Fig. 3b, c). These unusual current transitions may be attributed to the intracellular structures of microalgae. *Haematococcus* sp. cells possess highly complex intracellular architectures, including nucleus, chloroplast with multiplex membranes and prominent lipid bodies. These organelles are often intracellularly dominant. In fact, chloroplasts were shown to occupy the majority of the cell volume (Fig. 4a-c). Therefore, after injection into the cells, the tip of nanopipette may be localized near the organelle surface, resulting in ion migration hinderance and ionic current fluctuations. Further research involving the detailed analyses of the intracellular structures in microalgae should be performed to elucidate the precise mechanisms underlying these fluctuations.

## Conclusion

We developed an automated nanoinjection technique using nanopipettes for the efficient delivery of materials into green microalgae. This method offers a minimally invasive and effective alternative to existing delivery methods commonly used in microalgal research and can be applied across a broad range of eukaryotic microalgae species. Moreover, the quantitative delivery capability at the femtoliter scale provided by this technique enables precise and highly accurate evaluation of genetic transformation efficiencies in microalgae, paving the way for future advancements in microalgal genetic engineering.

## Supplementary Information

Below is the link to the electronic supplementary material.

Supplementary file1 (DOCX 587 KB)

## Data Availability

No datasets were generated or analysed during the current study.

## References

[CR1] Arakaki A, Iwama D, Liang Y, Murakami N, Ishikura M, Tanaka T, Matsunaga T (2013) Glycosylceramides from marine green microalga *Tetraselmis* sp. Phytochemistry 85:107–114 23089133 10.1016/j.phytochem.2012.09.006

[CR2] Chen Z, Zhu J, Chen Z, Du M, Yao R, Fu W, Lei A, Wang J (2022) High-throughput sequencing revealed low-efficacy genome editing using Cas9 RNPs electroporation and single-celled microinjection provided an alternative to deliver CRISPR reagents into Euglena gracilis. Plant Biotechnol J 20:2048–2050 36036114 10.1111/pbi.13915 PMC9616521

[CR3] Chen W, Li T, Du S, Chen H, Wang Q (2023) Microalgal polyunsaturated fatty acids: Hotspots and production techniques. Front Bioeng Biotechnol 11:1146881 37064250 10.3389/fbioe.2023.1146881 PMC10102661

[CR4] Frost LS, Ippen-Ihler K, Skurray RA (1994) Analysis of the sequence and gene products of the transfer region of the F sex factor. Microbiol Rev 58:162–210 7915817 10.1128/mr.58.2.162-210.1994 PMC372961

[CR5] Garza EA, Bielinski VA, Espinoza JL, Orlandi K, Alfaro JR, Bolt TM, Beeri K, Weyman PD, Dupont CL (2023) Validating a promoter library for application in plasmid-based diatom genetic engineering. ACS Synth Biol 12:3215–3228 37857380 10.1021/acssynbio.3c00163 PMC10661051

[CR6] Heiat M, Aghamollaei H, Hoseinei SM, Larki RA, Yari K (2012) Optimization of plasmid electrotransformation into *Escherichia coli* using Taguchi statistical method. Afr J Biotechnol 11:7603–7608

[CR7] Huang PW, Wang LR, Geng SS, Ye C, Sun XM, Huang H (2021) Strategies for enhancing terpenoids accumulation in microalgae. Appl Microbiol Biotechnol 105:4919–4930 34125275 10.1007/s00253-021-11368-x

[CR8] Jeon K, Suresh A, Kim YC (2013) Highly efficient molecular delivery into *Chlamydomonas reinhardtii* by electroporation. Korean J Chem Eng 30:1626–1630

[CR9] Karas BJ, Diner RE, Lefebvre SC, Mcquaid J, Phillips AP, Noddings CM, Brunson JK, Valas RE, Deerinck TJ, Jablanovic J, Gillard JT, Beeri K, Ellisman MH, Glass JI, Hutchison CA, Smith HO, Venter JC, Allen AE, Dupont CL, Weyman PD (2015) Designer diatom episomes delivered by bacterial conjugation. Nat Commun 6:6925 25897682 10.1038/ncomms7925 PMC4411287

[CR10] Kassaw TK, Paton AJ, Peers G (2022) Episome-based gene expression modulation platform in the model diatom *Phaeodactylum tricornutum*. ACS Synth Biol 11:191–204 35015507 10.1021/acssynbio.1c00367

[CR11] Kumar M, Jeon J, Choi J, Kim SR (2018) Rapid and efficient genetic transformation of the green microalga *Chlorella vulgaris.* J Appl Phycol 30:1735–1745

[CR12] Maeda Y, Tsuru Y, Matsumoto N, Nonoyama T, Yoshino T, Matsumoto M, Tanaka T (2021) Prostaglandin production by the microalga with heterologous expression of cyclooxygenase. Biotechnol Bioeng 118:2734–2743 33851720 10.1002/bit.27792

[CR13] Mularczyk M, Michalak I, Marycz K (2020) Astaxanthin and other nutrients from *Haematococcus pluvialis*-multifunctional applications. Mar Drugs 18:459 32906619 10.3390/md18090459 PMC7551667

[CR14] Muñoz CF, De Jaeger L, Sturme M, Lip K, Olijslager J, Springer J, Wolbert E, Martens D, Eggink G, Weusthuis R, Wijffels R (2018) Improved DNA/protein delivery in microalgae - A simple and reliable method for the prediction of optimal electroporation settings. Algal Res 33:448–455

[CR15] Run C, Fang L, Fan J, Fan C, Luo Y, Hu Z, Li Y (2016) Stable nuclear transformation of the industrial alga *Chlorella pyrenoidosa*. Algal Res 17:196–201

[CR16] Shah MM, Liang Y, Cheng JJ, Daroc M (2016) Astaxanthin-producing green Microalga *Haematococcus pluvialis*: from single cell to high value commercial products. Front Plant Sci 7:531 27200009 10.3389/fpls.2016.00531 PMC4848535

[CR17] Sreenikethanam A, Raj S, Gugulothu P, Bajhaiya AK (2022) Genetic engineering of microalgae for secondary metabolite production: recent developments, challenges, and future prospects. Front Bioeng Biotechnol 10:836056 35402414 10.3389/fbioe.2022.836056 PMC8984019

[CR18] Stanley J, Lohith A, Debiaso L, Wang K, Ton M, Cui W, Gu W, Fu A, Pourmand N (2023) High throughput isolation of RNA from single-cells within an intact tissue for spatial and temporal sequencing a reality. PLoS ONE 18:e0289279 37527243 10.1371/journal.pone.0289279 PMC10393160

[CR19] Tanaka T, Maeda Y, Suhaimi N, Tsuneoka C, Nonoyama T, Yoshino T, Kato N, Lauersen KJ (2021) Intron-mediated enhancement of transgene expression in the oleaginous diatom *Fistulifera solaris* towards bisabolene production. Algal Res 57:102345

[CR20] Wang L, Yang L, Wen X, Chen Z, Liang Q, Li J, Wang W (2019) Rapid and high efficiency transformation of *Chlamydomonas reinhardtii* by square-wave electroporation. Biosci Rep 39:BSR20181210 30530569 10.1042/BSR20181210 PMC6328877

[CR21] Wang M, Ye X, Bi H, Shen Z (2024) Microalgae biofuels: illuminating the path to a sustainable future amidst challenges and opportunities. Biotechnol Biofuels Bioprod 17:10 38254224 10.1186/s13068-024-02461-0 PMC10804497

[CR22] Wichmann J, Baier T, Wentnagel E, Lauersen KJ, Kruse O (2018) Tailored carbon partitioning for phototrophic production of (E)-α-bisabolene from the green microalga *Chlamydomonas reinhardtii*. Metab Eng 45:211–222 29258965 10.1016/j.ymben.2017.12.010

