# Supplementary material for: Electroosmotic Flow-Based Nanoinjection Technique Using a Nanopipette for Green Microalgae
Source: Mar Biotechnol (NY). 2025 Jul 1;27(4):108. doi: 10.1007/s10126-025-10487-0 (PMC12213965; doi:10.1007/s10126-025-10487-0)
Supplement: Supplementary file 1 — Supplementary file1 (DOCX 587 KB) [file 10126_2025_10487_MOESM1_ESM.docx]

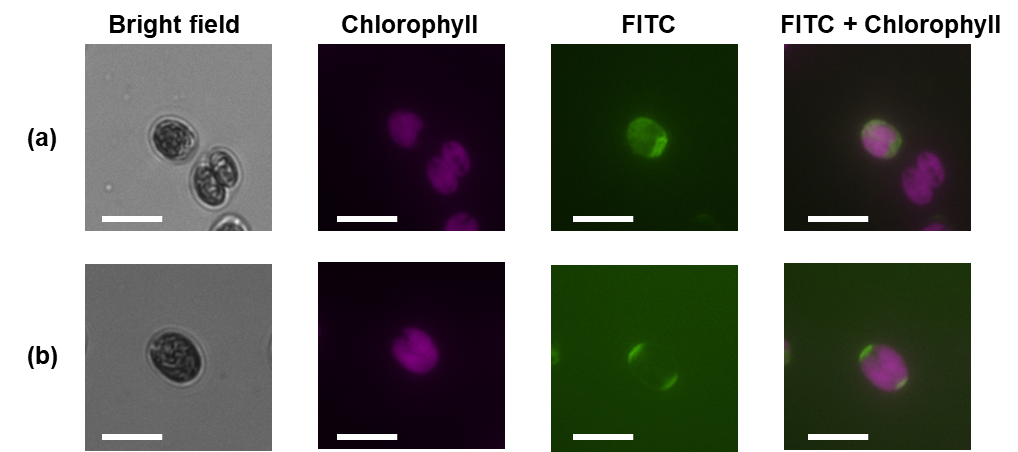


**Fig. S1**　(a-b) Microscopic images of *Tetraselmis* sp. strain NKG400013 following the injection of FITC-dextran. Scale bars = 20 μm. Green: FITC, Magenta: Chlorophyll autofluorescence

**
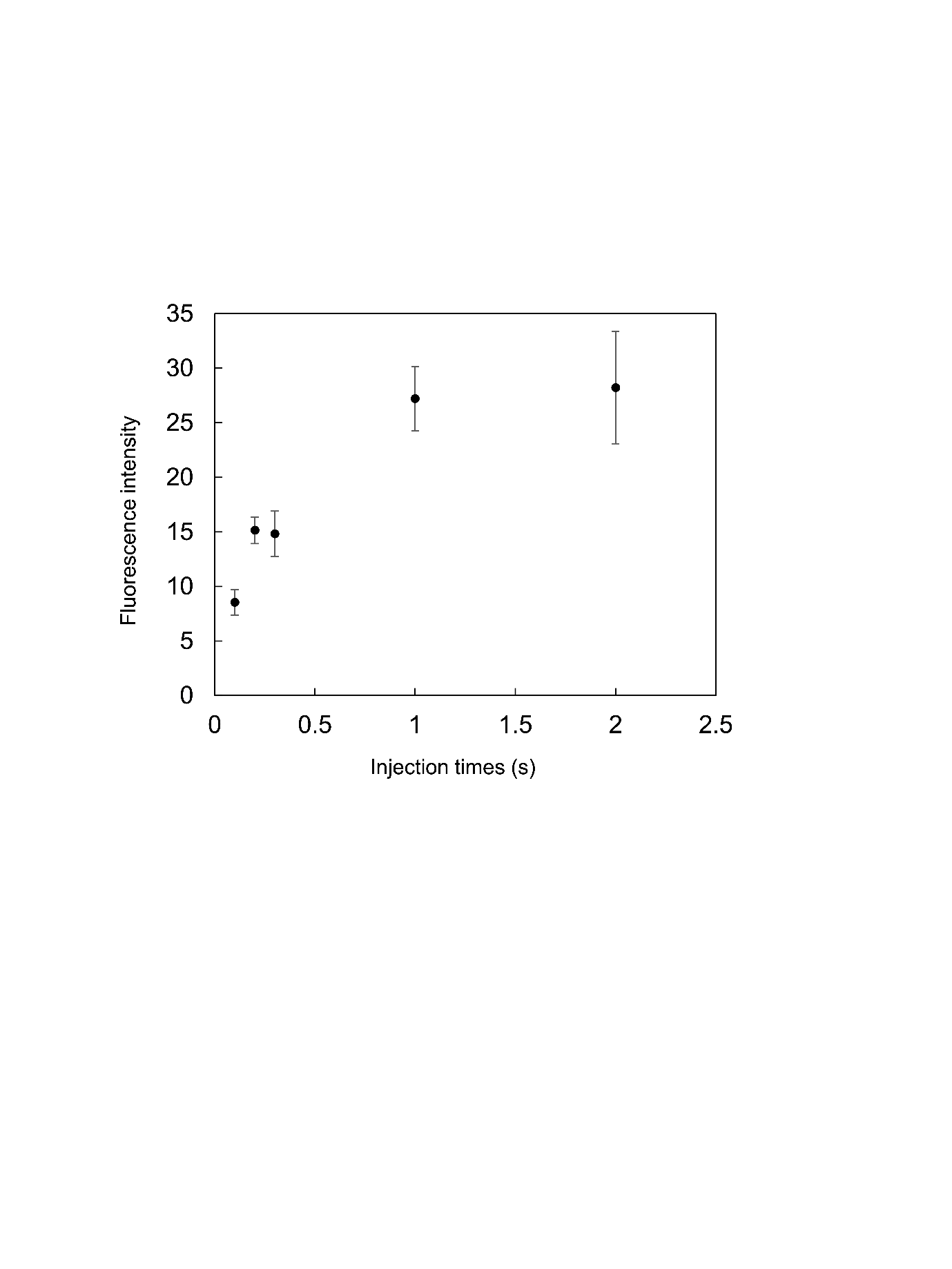
**

**Fig. S2** Correlation between fluorescence intensities of FITC-dextran-injected mammalian cells and injection durations
